# Reconstructing patterns of domestication in reindeer using 3D muscle attachment areas

**DOI:** 10.1007/s12520-023-01910-5

**Published:** 2023-12-29

**Authors:** Christina Siali, Sirpa Niinimäki, Katerina Harvati, Fotios Alexandros Karakostis

**Affiliations:** 1 Palaeoanthropology, Senckenberg Centre for Human Evolution and Palaeoenvironment, Institute for Archaeological Sciences, Eberhard Karls University of Tübingen, Tubingen, Germany; 2 Archaeology, History, Culture and Communication Studies, Faculty of Humanities, University of Oulu, Oulu, Finland; 3 DFG Centre of Advanced Studies ‘Words, Bones, Genes, Tools’, Eberhard Karls University of Tübingen, Tubingen, Germany

**Keywords:** Reindeer domestication, Activity reconstruction, Entheseal patterns, Animal management

## Abstract

The use of reindeer has been a crucial element in the subsistence strategies of past Arctic and Subarctic populations. However, the spatiotemporal occurrence of systematic herding practices has been difficult to identify in the bioarchaeological record. To address this research gap, this study proposes a new virtual anthropological approach for reconstructing habitual physical activity in reindeer, relying on the protocols of the “Validated Entheses based Reconstruction of Activity” (VERA) method. Following blind analytical procedures, we focused on eight muscle attachment sites (“entheses”) in 36 reindeer free ranging in the wild, 21 specimens in captivity (zoo), and eight racing reindeer (habitual runners). Importantly, our analyses accounted for the effects of variation by subspecies, sex, age, and estimated body size. Our results showed clear differences across activity groups, leading to the development of discriminant function equations with cross-validated accuracies ranging from approximately 88 to 100%. The reliability of our functions was additionally confirmed using a blind test involving six zoo individuals not included in the initial dataset. Our findings support the use of the proposed approach for identifying domestication-related activities in zooarchaeological contexts, introducing a valuable tool for locating suspected domestication hotspots and elucidating the nature of past human-reindeer interactions.

**Supplementary Information:**

The online version contains supplementary material available at 10.1007/s12520-023-01910-5.

## Introduction

Domestication of animals and plants has been a significant driving force in the socio-economic dynamics of human societies (Childe 1928; Roberts 2014; Zeder 2015). Domestication is a gradual, mutualistic process where animals influence human behaviour, and in turn, humans significantly regulate the animals’ activities, eventually leading to behavioural changes (Zeder 2015). Long-term animal management can result in full domestication of a species, accompanied by morphological changes and husbandry signals (Zeder 2011; Dobney et al. 2013). Early domestication practices can be detected with evidence such as demographic profiles (as is the case with sheep and goats; Zeder 2006; Peters et al. 2013) or evidence of corralling (Zeder 2006; Gaunitz et al. 2018).

In the case of reindeer, the detection of domestication and management related practices is challenged by the variable approaches to their management. Reindeer management ranges from nomadic or transhumant lifestyles involving large scale herding (Anderson et al. 2019) to smaller scale and controlled mobility herding (Aronsson 1991; Pelletier et al. 2020). Reindeer played a crucial role in the lives of Arctic and subarctic populations in terms of both economic and cultural significance (Helskog and Indrelid 2011; Anderson et al. 2019). Archaeologically, one of the first recorded instances of systematic human-reindeer interactions are found at the I͡Arte 6 site (Iron Age, Siberia; Nomokonova et al. 2018). The various lines of evidence produced from the site (Nomokonova et al. 2018; Anderson et al. 2019) suggest various types of interactions, from hunting to small-scale herding. These findings, however, do not suggest a high level of dependency comparable to the ethnographic examples observed in Nenets reindeer herders (see discussion, Anderson et al. 2019). In Fennoscandia, systematic herding appears in the Late Iron Age as evidenced by stone fences, hearths, reindeer milking locations, and bone deposits (Aronsson 1991). Currently, various indigenous populations in Siberia and Fennoscandia manage a large number of reindeer, also allowing them to roam freely throughout the year (Willerslev et al. 2015; Niinimäki and Salmi 2016). These varying intensities and strategies of reindeer management across ethnic groups, as well as through time and space (Aronsson 1991; Pelletier et al. 2020), create difficulties in identifying domestication-related practices (such as herding) based on contextual bioarchaeological evidence (i.e., skeletal remains of reindeer).

Reindeer are one of the few animals in a close relationship with humans that are still in the process of domestication (i.e., “semi-domesticated”) and have undergone limited morphological changes (Syroechkovski 2000; Pelletier et al. 2020). Therefore, identifying domestication markers in their skeletal remains is challenging (Pelletier et al. 2020). Despite these challenges, previous zooarchaeological research on reindeer has provided valuable insights into their habitual physical behaviour based on various skeletal activity markers, including three-dimensional (3D) morphometric analyses of long bones (Pelletier et al. 2020, 2022), cross-sectional properties of long bones and metapodials (Pelletier et al. 2021; Niinimäki and Salmi 2021; Niinimäki et al. 2021), and entheseal changes and paleopathology (Niinimäki and Salmi 2016; Salmi et al. 2020). For instance, recent research by Salmi and Niinimäki (2021) reconstructed reindeer feeding behaviour in archaeological samples based on macroscopically evaluated changes in muscle attachment bone sites. The findings of these previous studies suggested that activity reconstruction is a promising avenue for inferring domestication-related processes in the archaeological record, particularly in the early stages of animal domestication where animals have undergone little to no morphological changes (e.g., Salmi and Niinimäki 2021).

Entheses are defined as the areas on the bone’s surface where muscles or ligaments attach (e.g., Benjamin et al. 2002). Given that they comprise the only archaeologically surviving remnant of the human musculotendinous unit, they have been routinely used for reconstructing habitual muscle use and activity in past human populations (e.g., Schrader 2019). However, the reliability of this practice (Villotte 2006; Niinimäki and Salmi 2016; Wilczak et al. 2017; Bindé et al. 2019) has been repeatedly questioned (e.g.,Alioto 2015; Foster et al. 2014), mainly due to the low repeatability of most traditional visual scoring approaches and the reported lack of supportive experimental evidence that physical activity indeed has a significant effect on entheseal surface morphology (Zumwalt 2006; Rabey et al. 2015; Wallace et al. 2017). Recently, however, one of us (FAK) developed the “Validated Entheses based Reconstruction of Activity” (VERA) methodology (see Karakostis and Lorenzo 2016; Karakostis & Harvati 2021; Karakostis 2022 and references therein). This new approach addressed the main controversies in entheseal research by recording the 3D surface area of entheses based on precise delineation and measurement protocols (e.g., Karakostis & Lorenzo 2016; Karakostis et al. 2017, 2018, 2021), accompanied by rigorous multivariate statistical analyses (see extensive review in Karakostis 2022). The key benefit of this method is that it can identify patterns of habitual coordination among different muscles, providing a more solid and comprehensive basis for linking skeletal morphology with specific physical behaviours. Importantly, the reliability of VERA has been confirmed using human skeletons with extensively documented long-term occupational activities (Karakostis et al. 2017; Karakostis & Hotz 2022). Furthermore, VERA has been experimentally validated in several laboratory studies by Karakostis et al. (2019a, ba; 2019a, b; Karakostis and Wallace 2023) and Castro et al. (2022), focusing on diverse animal species (rats, mice, and turkeys) and activity regimes (e.g., uphill or downhill running and climbing). Despite these promising experimental finds involving laboratory animals, VERA has never been tested or applied on skeletal remains of larger-bodied animal species of interest for bioarchaeology (either reference collections or zooarchaeological samples).

In this framework, this study represents the first zooarchaeological application of the method VERA, aiming to test its applicability on a modern sample of reindeer with documented life histories, which consists of racing, zoo, and free ranging reindeer (Niinimäki and Salmi 2016; Pelletier et al. 2020, 2021, 2022; Salmi et al. 2020; Niinimäki et al. 2021; Salmi and Niinimäki 2021). By targeting the reconstruction of specific habitual movements of reindeer, including systematic running, or feeding via digging through the snow for lichen, we opt to develop and evaluate a reliable 3D entheses-based protocol for recognizing domestication-related activities in reindeer.

## Materials and methods

### Sampling strategy

The sample of this study is composed of 65 adult reindeer individuals from three different activity groups: free ranging, zoo, and racing reindeer (Niinimäki and Salmi 2016; Pelletier et al. 2020, 2021, 2022; Salmi et al. 2020; Niinimäki et al. 2021; Salmi and Niinimäki 2021). To account for the effects of age, only adult specimens with fused epiphyses were analysed. The free ranging group consists of 19 females and 17 males, the racing group consists of eight males, while the zoo group comprises of 13 females and eight males. The majority of these reindeer skeletons are curated at the University of Oulu's Biodiversity Unit (Table 1) and have been used in multiple studies addressing reindeer activity (Niinimäki and Salmi 2016; Pelletier et al. 2020, 2021, 2022; Salmi et al. 2020; Niinimäki et al. 2021; Salmi and Niinimäki 2021). Finally, six out of 21 zoo specimens derive from the State Collection in Munich (see Acknowledgements). It should be clarified that the final sample size varied across analyses due to preservation issues (see Table 1).

**Table 1:** Sample sizes per group and bone element divided by sex. The humeri and radio-ulnae in the sample correspond to the same individual animals. The sample size decreased in the case of the radio-ulna due to preservation issues in certain individuals

Group	Humerus		Total	Radio-ulna		Total
Free ranging	19 Females	17 Males	36	17 Females	13 Males	30
Racing	**-**	8 Males	8	-	8 Males	8
Zoo	9 Females	6 Males	15	7 Females	5 Males	12
Munich Zoo (blind sample)	4 Females	2 Males	6	-	-	-
Total sum			**65**			**51**

The sample contains two subspecies of reindeer: 30 *Rangifer tarandus* (also known as mountain reindeer), 35 *Rangifer tarandus fennicus* (also known as forest reindeer), and one hybrid of these (Niinimäki and Salmi 2016; Salmi et al. 2020; Salmi and Niinimäki 2021). In comparison to mountain reindeer *R.t.tarandus*, forest reindeer *R.t.fennicus* are reported to have a slimmer anatomy and relatively longer limbs (Niinimäki and Salmi 2016; Pelletier et al. 2020). In this study, we confirmed that subspecies variation did not considerably influence our findings (see Online resources 7 & 8).

### Entheseal selection and biomechanical hypothesis

During winter and spring, our free ranging reindeer of the subspecies *R.t.tarandus* roamed freely, feeding on natural pastures by digging through the snow for lichen, an activity that took about 7–8 h per day (Niinimäki and Salmi 2016; Salmi et al. 2020; Salmi and Niinimäki 2021). Then, during summer and autumn, they were herded up for the earmarking of new calves and the selection of individuals for slaughter. Our reindeer of the subspecies *R.t.fennicus*, on the other hand, were not managed, roaming freely throughout the year (Niinimäki and Salmi 2016; Salmi et al. 2020; Salmi and Niinimäki 2021). Similarly, to the free ranging ones, the zoo reindeer comprised both *R. t. fennicus* and *R. t. tarandus* subspecies (as well as one hybrid of the two subspecies), all deriving from Oulu University Zoo and Ranua zoo (*n* = 1). The reindeer in Oulu University Zoo were corralled in a 570-m^2^ enclosure with flat terrain (Niinimäki and Salmi 2016; Salmi et al. 2020; Salmi and Niinimäki 2021), while the six reindeer from the Munich's State Zoological Collection spent their lives confined within the zoos of Nuremberg and Helsinki. Finally, the racing reindeer had been initially selected from herds of free ranging reindeer and thus spent 2 years of their lives in natural pastures, digging in the snow for lichen. Due to the present practice of selecting only male individuals for training, the racing group is composed entirely of male reindeer. Once selected, at around 3 years of age, they would spend the first year accustomed to a tether and pulling weight, running laps with the owner on a snow mobile. After the first year, they would spend the winter training for 3 h per week to improve their stamina and speed. During racing competitions, they would be harnessed to pull the driver (weighing 60–65 kg) forward (for more details, see Niinimäki and Salmi 2016; Salmi et al. 2020; Salmi and Niinimäki 2021).

**Fig. 1:**
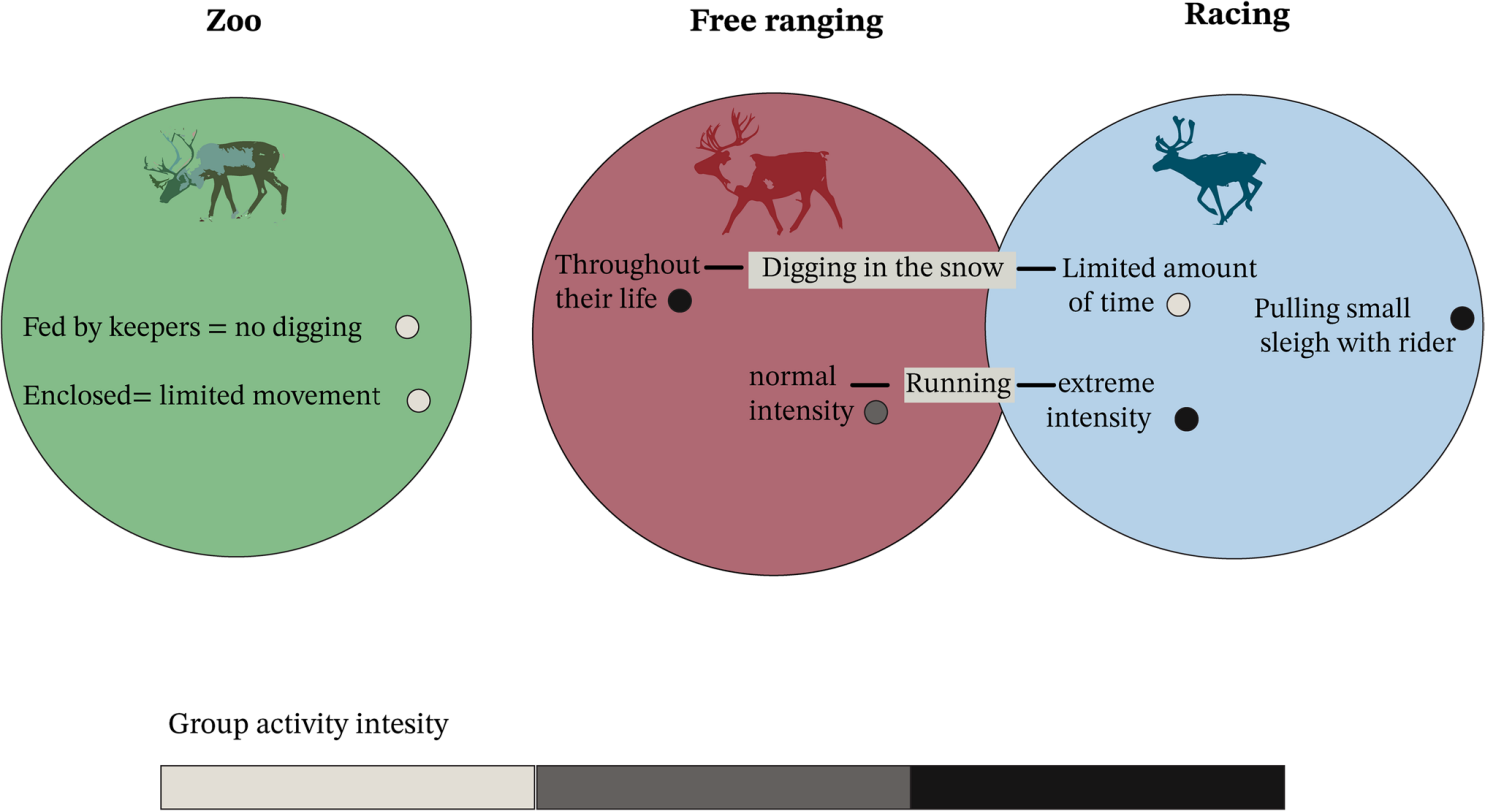
Schematic representation of the activities of the three reindeer groups: racing, free ranging, and zoo reindeer. The activities of the free ranging and racing groups overlap, but at varying intensities (for more details, see Niinimäki and Salmi 2016; Salmi et al. 2020). Activity group intensity is represented with the grey scale dots next to the activities. The darker the colour, the greater the intensity of activity indicated

The analysis includes three distinct activity groups that correspond to three different activity gradients. As deduced from their documented activity histories, the zoo reindeer were the least mobile of the three, whereas the free ranging reindeer presented a substantially greater mobility. Finally, the racing reindeer represent the most active group (figuratively speaking, the “athletes” of our sample). Racing reindeer are expected to have systematically run during racing competitions and practice, harnessed to pull the rider’s full weight (Niinimäki and Salmi 2016; Salmi et al. 2020; Salmi and Niinimäki 2021). It should be noted that although the racing reindeer were initially selected from free ranging herds as mentioned above, they would have dug in the snow only for the duration of one winter. More specifically, the reindeer fed next to their mothers (primary diggers) during their first year as calves, and by their third year, they were already being trained and fed by their keepers. Free ranging reindeer, on the other hand, are expected to have been highly mobile and digging in the snow for food. Lastly, the zoo reindeer were neither mobile nor digging (Fig. 1) (Niinimäki and Salmi 2016; Salmi et al. 2020; Salmi and Niinimäki 2021). Given the activities described, we chose to focus on the forelimbs, which have been shown to provide stability and endure biomechanical pressures, such as those experienced during reindeer migrations (Li et al. 2020). Additionally, forelimbs play a major role in weight-bearing (Niinimäki and Salmi 2016; Pelletier et al. 2020) in ungulates and are primarily used by reindeer for digging in the snow for lichen, an activity targeted in this study. In the absence of biomechanical data pertaining to the exact muscle synergies involved in the specific movements of reindeer (Wareing et al. 2011), we selected a set of ten humeral and radio-ulnar entheses that are expected to be involved in the physical tasks targeted in this study (Table 2). More specifically, the muscles selected are involved in extension and flexion of the shoulder joint, abduction and adduction of the forelimb, and extension of the elbow and digits (König et al. 2007; Wareing et al. 2011). In total, our selection included seven muscles of the humerus and three muscles and ligaments of the radio-ulna (Table 2). Following the VERA approach, we expect the entheseal patterns of each group to reflect muscle interactions associated with their habitually performed activities.

**Table 2:** Basic characteristics of the muscles and ligaments associated with the entheses analysed in this study. The term “Type” refers to whether the attachment site represents the origin or the insertion enthesis of each described muscle or ligament. The asterisk indicates muscles or ligaments that share an attachment site with others in our analyses (for more information, see in the text below)

Muscle/ ligament	Muscle function	Location / type	Reference
*Supraspinatus*	Flexion of the shoulder	Greater tubercle of the humerus / Insertion	Wareing et al. (2011)
*Subscapularis*	Flexion of the shoulder	Lesser tubercle of humerus / Insertion	Wareing et al. (2011)
Deltoideus*	Flexion of the shoulder	Deltoid tuberosity / Insertion	Wareing et al. (2011)
Teres Minor*	Flexion of the shoulder	Tricipital line of humerus / Insertion	Wareing et al. (2011)
*Infraspinatus*	Flexion and extension of the shoulder	Greater tubercle of the humerus / Insertion	Wareing et al. (2011)
*Triceps Brachii* (lateral head) *	Flexion of the elbow	Tricipital line of humerus / Origin	Wareing et al. (2011)
*Teres Major*	Flexion of the shoulder, adduction of the limb	Teres Major tuberosity (Medial surface of the humerus shaft) / Insertion	Wareing et al. (2011)
*Flexor digitorum profundus* (ulnar and radial portions)	Flexion of the digits	Radial portion: Lateral surface groove between the fused radius and ulna / Origin Ulnar portion: Medial surface of the olecranon of the ulna / Origin	Budras et al. (2003); König et al. (2007); Wareing et al. (2011)
Distal radiocarpal and medial collateral ligament*	Allows flexion and extension of the carpal joints/ shock absorber	Radial trochlea and styloid process of the radio-ulna	Budras et al. (2003); König et al. (2007); Wareing et al. (2011)

In certain cases (denoted in Table 2 by an asterisk), different muscles attach to the same distinct bone structure (e.g., a tuberosity). In particular, the muscles *triceps brachii*, *teres minor*, and *deltoideus* (hereafter collectively referred to as “deltoideus”) insert into the deltoid tuberosity and the tricipital line, which together form a rather uniform and continuous structure in reindeer. Respectively, the distal radiocarpal and medial collateral ligaments (hereafter collectively referred to as “radio-ulnar ligament”) share a common attachment region in the radio-ulna (Table 1). Following standard VERA practice (e.g., see Karakostis and Lorenzo 2016; Karakostis and Harvati 2021; Karakostis 2022), given that the exact footprint of each tendon within the same dry bone structure was not perfectly distinguishable across all specimens, the entire bone structure was measured and used to represent all associated muscles together. Consequently, the ten structures analysed in this study (eight muscles and two ligaments) correspond to a total of eight entheseal surfaces measured. Considering that the above muscles attaching to the same structure are closely synergistic and systematically activate together for the activities investigated in this study (Table 1), we believe that our functional interpretations were not considerably affected by this lack of distinction (see Discussion).

### 3D measurement

The reindeer curated in the biodiversity unit of the University of Oulu were scanned using a medical CT scanner (Somatom Definition Flash, Siemens Healthcare, Forcheim, Germany). The humeri and radio-ulnae used in this study were scanned at 120 kVp, 700 eff. mAs, 0.5-s rotation time, 0.6-mm slice thickness and increment, B70f reconstruction kernel, 140-mm reconstruction diameter, 0.35 pitch, and 128 × 0.6 collimation (Pelletier et al. 2020). Subsequently, 3D surface models were developed and extracted in STL format for further analysis. The entheseal measurements were conducted using the VERA protocols for delineating and measuring entheses (Karakostis and Lorenzo 2016), whose steps have been extensively described and illustrated in previous studies (Karakostis et al. 2019a; 2019b; Castro et al. 2022; Karakostis and Harvati 2021; Karakostis 2022). In brief, the 3D models of the Reindeer humeri and radio-ulnae were inserted into the open-access software MeshLab (version 2020.12, CNR inc., Pisa), where the attachment sites (entheses) of the selected muscles were identified (Table 1). The entheseal surface areas were then visually examined across 360° in terms of surface elevation and complexity, relying both on the unfiltered mesh and on various imaging filters that colour-map the surface based on calculations of discrete curvatures and surface depth (for more details on the exact steps, see reviews by Karakostis and Harvati 2021; Karakostis 2022). The borders of the entheses were delineated, and their mesh surface areas were calculated in mm^2^, as per the VERA protocols for either human (e.g., Karakostis & Lorenzo 2016; Kunze et al. 2022; Bousquié et al. 2022) or animal (Castro et al. 2022; Karakostis et al. 2019a; 2019b; 2023) skeletal remains.

The intra- and inter-observer measuring precision (repeatability) of this approach has been demonstrated in several previous studies (Karakostis and Lorenzo 2016; Castro et al. 2022; Kunze et al. 2022). Here, intra-observer repeatability was further confirmed by performing a precision test that involved all entheses of six randomly selected reindeer. All measurements were taken three times, with an interval of 2 days in-between repetitions. Importantly, to further eliminate observer bias, this study’s measurements and analyses were carried out by the lead author (CS) under blind analytical conditions (i.e., the sex and activity group of each analysed reindeer were not known to the observer). This documentation was only available to authors FAK and SN and was only disclosed to the first author after all entheseal measurements were completed.

Additionally, for the purpose of the discriminant analyses (see subsection below), the measured areas (in mm^2^) were size-adjusted using the geometric mean approach (Karakostis 2022). Each entheseal area measurement was divided by the geometric mean of all entheseal measurements from the same individual (Friehauf et al. 2013). Weight information was available for 51 out 65 individuals (Niinimäki and Salmi 2016; Niinimäki et al. 2021), whereas for the remaining 14 individuals, the weight was estimated based on humeral linear measurements following Puputti and Niskanen (2008).

### Statistical analyses

**Fig. 2:**
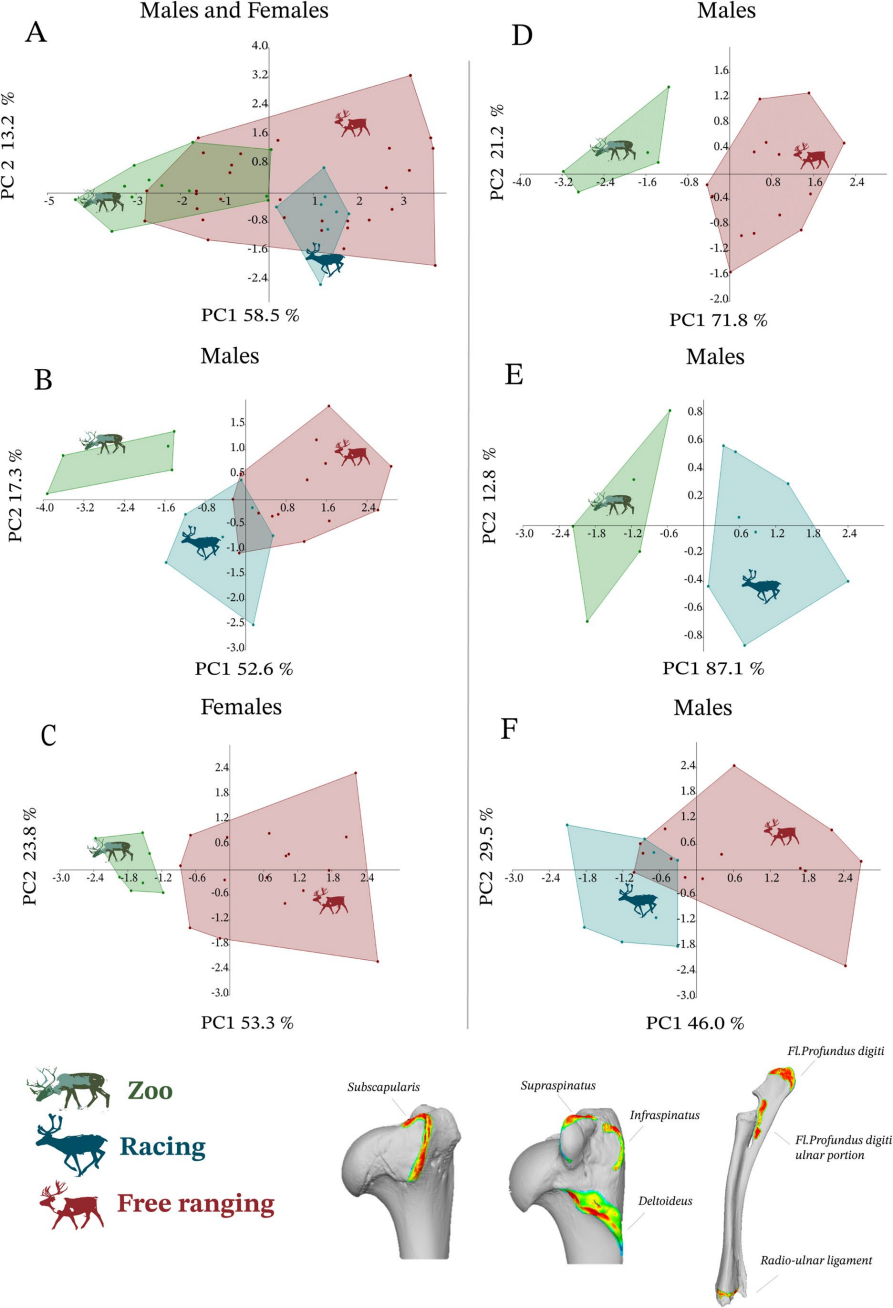
Plots of principal component analyses (PCAs) conducted in this study: **A** Plot of the PCA involving all females and males from all three activity groups. **B** Plot of the PCA based on the males of all three activity groups. **C** Plot of the PCA based on all females present in our sample, i.e., free ranging and zoo groups (note that racing is an all-male sample — see introduction); **D** Plot of the PCA on the zoo and free ranging males **E**) Plot of the PCA on racing and zoo males; **F** Plot of the PCA on the males of the free ranging and racing groups. Factor loadings and eigenvalues for all PCAs of Fig. 2 are listed in Online resource 9. The bottom right of the figure displays the selected entheses delineated in Meshlab, using VERA protocols

The entheseal 3D measurements were subjected to a series of principal component analyses (PCAs) and discriminant function analyses (DFAs). To explore variation across individuals without assigning any activity groups a priori, we ran a series of PCAs involving multiple possible combinations of the muscle entheses used in this study (Table 2). In each PCA, we ensured that the number of variables did not surpass the sample size requirements (at least five cases per variable, e.g., see Field 2018). Plots of PCAs revealing functionally meaningful variation across groups are presented below (in Results). To assess whether body size influenced the observed multivariate patterns among entheses, we used the Spearman’s rank correlation coefficient to test the association between the PC1 scores of selective PCAs (Fig. 2) and estimated (or recorded) body weight (in kg).

Subsequently, DFAs were conducted based on various combinations of entheses and sexes (both sexes pooled together and separately) (see Online resources 10 & 11). These included 18 DFAs using the 3D measurements (mm^2^), encompassing eight analyses on all individuals (both sexes) and 10 sex-specific ones (either male- or female-only samples). Finally, to evaluate whether controlling for the effects of size can further increase the accuracy of our discriminant functions, an additional 18 DFAs were conducted involving values adjusted for size using the geometric mean approach (see in subsection above).

Prior to these analyses, we confirmed that all necessary statistical assumptions were met. For the DFA, these included variance–covariance matrix homogeneity, which was evaluated using the Box's M test (Field 2018). Out of 36 DFAs conducted in this analysis in total (see below), only eight provided a significant Box’s M *p*-value (> 0.05), indicating a potential violation. To address this issue, we re-run these eight analyses using separate group covariance matrices (e.g., Field 2018). Given that the latter showed similar results, this apparent statistical violation does not seem to have considerably affected our results (e.g., see Karakostis et al. 2018). Other assumptions included the absence of multivariate outliers (confirmed based on Mahalanobis distances; see Field 2018), linearity among variables, and minimum sample size requirements (i.e., PCA assumes at least five cases per variable, while DFA requires more cases than variables within each factor level; e.g., see Field 2018). PCA was conducted using the PAST software 4.03 (Hammer 2001), whereas DFA was performed in SPSS 21 (IBM SPSS Inc.) The PCAs were performed on a correlation matrix because of varying ranges across variables (Field 2018). The number of components plotted was determined by the standard scree plot technique (e.g., Field 2018).

Furthermore, we developed discriminant function equations for predicting entheseal activity group membership based either on entheseal measurements or size-adjusted values (Field 2018; Karakostis et al. 2018; Karakostis and Harvati 2021). For this purpose, each DFA’s unstandardized coefficients, constant, and group centroids are listed in Online resources 10 and 11. Each function’s accuracy rate was estimated both before and after cross-validation, following a “leave-one-out classification” procedure (e.g., Landau and Everitt 2004; Field 2018).

The reliability of the most successful discriminant functions was further evaluated using a blind test, focusing on six reindeer deriving from the Prague (*n* = 5) and Helsinki (*n* = 1) zoos. Our goal was to assess if the activity group of these individuals could be reliably predicted, considering that they were not parts of our sample and they originate from a different geographical region. This additional “blind test sample” was scanned at the Munich State Collection using an Artec Space Spider scanner (Artec Inc., Luxembourg) with a measuring accuracy of up to 50 μm. Since only the humeral entheseal 3D surfaces were available to us, the blind test focused on the entheses of muscles *supraspinatus*, *subscapularis*, *infraspinatus*, and *deltoideus*. In the original sample, this muscle combination was found to present considerable differences across groups (see “Results,” subsection “Discriminant function analyses”). These humeral entheseal surfaces of the six “Munich reindeer” were measured, and their activity group membership was assessed using a series of our developed DFAs (see below and protocol described in Online resources pg. 1–4).

## Results

### Principal component analyses (PCAs)

Figure 2 presents six of our PCAs, either presenting both sexes together (Fig. 2A) or females and males separately (Fig. 2B–E). Overall, the clearest activity-related variation was observed when comparing groups in pairs (rather than plotting all three groups together) and focusing measurements of each sex separately. Racing and zoo animals, representing two extremely opposing intensities of physical activity (Fig. 1), showed the clearest differences across analyses (e.g., Fig. 2B). For the analyses comparing free ranging to either the zoo (Fig. 2D and E) or the racing (Fig. 2C) reindeer, the clearest activity-related differences were found when analysing females and males separately (Fig. 2A vs B–F). Among pairwise analyses, the only PCA plot in which the activity groups partly overlapped was the one involving free ranging and racing reindeer. Nevertheless, even in that plot, there was a clear propensity for racing reindeer to plot on the negative axis of PC1 (46% of variance), with most free ranging specimens presenting positive values (Fig. 2C). The PCAs below show pooled data for *R.t. tarandus* and *R.t. fennicus* subspecies in the free ranging and zoo groups (racing consists of *R.t. tarandus* individuals only). Additional analyses confirmed that subspecies variation does not considerably affect the observed activity patterns (see Discussion & Online resources 7 & 8).

### Discriminant function analyses (DFAs)

DFA was able to further highlight the observed entheseal variation between the three activity groups. These differences were most distinct when comparing groups in pairs and, in most cases, when focusing on each sex separately. More specifically, reflecting the PCA trends, racing and zoo reindeer were correctly classified in 100% of the cases both before and after cross-validation, and both when using unadjusted 3D entheseal measurements or size-adjusted values (Table 3; Online resources 10 & 11). The second most effective comparison was that of free ranging versus zoo animals, which achieved a cross-validated classification accuracy of 88% when both sexes were pooled together. Nevertheless, these classification rates significantly increased when males (100%) and females (91%) were analysed separately based on unadjusted entheseal measurements. Finally, racing and free ranging reindeer were correctly classified in 88% of the cases when using size-adjusted values to compare between the racing specimens (all males) and pooling all free ranging individuals (males and females combined).

**Table 3:** Classification accuracy rates of the most effective DFAs. For each group, the exact number of correctly classified individuals are indicated in bold under the classification percentages. The groups either involve males and females together or females and males separately (see categories in bold). All classifications presented are cross-validated. The asterisk denotes the use of size-adjusted values (instead of unadjusted measurements). The statistics of the associated DFAs can be found in Online resources Tables 5 to 9, 10, and 11

DFA classification percentages				
Group combinations	Total	Free ranging	Racing	Zoo
**Females and males together**				
All groups	70.0%*	56.7%	87.5%	91.7%
		**17/30**	**7/8**	**11/12**
Free ranging/ zoo	88.1%	86.7%		91.7%
		**26/30**		**11/12**
Racing/ zoo	100.0%		100.0%	100.0%
			**8/8**	**12/12**
Racing / free ranging	88.1%*	91.2%	75.0%	
		**31/34**	**6/8**	
**Females and males separately**				
All groups – males	77.8%	78.6%	75.2%	80.0%
		**11/14**	**6/8**	**4/5**
Free ranging/ zoo – males	100.0%	100.0%		100.0%
		**14/14**		**5/5**
Free ranging/ zoo – females	91.3%	87.0%		100.0%
		**14/16**		**7/7**
Racing / zoo – males	100.0%		100.0%	100.0%
			**8/8**	**5/5**
Racing / free ranging – males	80.0%*	64.3%	100.0%	
		**9/14**	**8/8**	

### Multivariate entheseal patterns

The above DFAs and PCAs revealed similar and distinct patterns of activity group separation based on specific group and muscle combinations. For all entheses, the more active groups (i.e., free ranging and racing) appear to have larger values than the less mobile zoo animals. Among entheses, the relative proportions of teres major and the distal radio-ulnar ligament were the most impactful in DFAs and PCAs that included free ranging reindeer (Online resources 9, 10 & 11). On the other hand, the entheses of muscles deltoideus, subscapularis, and teres major were the most influential in most analyses involving the racing group. Finally, when comparing free ranging and racing reindeer, focusing on the proportions expressed from the muscles involved in the propelling movement of the shoulder, such as the infraspinatus, subscapularis, supraspinatus, and deltoideus, better captured the differences among these two groups.

### Correlation with weight

We addressed the influence of weight by testing the correlation between weight and PC1 scores of selective PCAs (Fig. 2; Table 4). When pooling females and males of all groups together, the Spearman’s *ρ* value obtained indicated a significant (*p*-value < 0.01) and strong (> 0.60) positive relationship (Tabachnick and Fidell 1989) (Table 4). Nevertheless, when considering only the male sample (excluding the females), the correlation with weight appeared to be non-significant, indicating that sexual dimorphism in body weight has likely influenced the results of the first correlation test (Table 4). The same applied for the two correlation tests involving either free ranging and zoo or free ranging and racing males, respectively (Table 4). Nevertheless, this was not the case for the sample including racing and zoo males, for which a significant positive correlation was found (Tabachnick and Fidell 1989) (Table 4). Finally, the correlation test focusing on the PC1 values of free ranging and zoo females showed a significant *p*-value and a strong positive correlation coefficient (Tabachnick and Fidell 1989).

**Table 4:** Non-parametric Spearman’s corelation tests’ *ρ* and *p* values. The tests were performed using the individuals’ PC1 scores (PCAs shown in Fig. 2) and corresponding weight (estimated or recorded). All significant correlations are shown in bold

	Spearman’s *ρ*	*P*-value
All groups (both sexes)	0.76	** < 0.01**
All groups’ males	0.18	0.36
Racing and zoo males	0.57	**0.03**
Free ranging and zoo males	0.43	0.19
Free ranging and zoo females	0.67	** < 0.01**
Free ranging and racing males	-0.18	0.22

### Blind test

To further strengthen the applicability of the method proposed in this paper, we conducted a blind test on six zoo reindeer from the Munich Collection (SNSB – See Acknowledgements). To assess their group affinity, we relied on the discriminant equations produced in this study, which were performed in a specific sequence (Table 5) following our proposed step-by-step protocol (Online resources pg. 1–4). The results were consistent with the fact that these six Munich reindeer had the same sedentary lifestyle as the original sample’s zoo reindeer from Finland (Niinimäki and Salmi 2016; Salmi et al. 2020). More specifically, almost all DFA equations classified the six specimens with the “zoo” group. The only exception was the DFA equation involving free ranging and zoo females, which misclassified one animal (Table 5).

**Table 5:** Blind test involving six reindeer from Munich and the use of the DFA function equations developed in this study for entheses of the humerus (see “Materials and methods”). The blind test followed the steps of the protocol outlined in Online resources pg. 1–4 (upper part of table, in bold). The test included some additional classifications outside of the protocol (lower part of table, in bold)

Protocol Steps / Groups	DFA Classification %	Classification of the Munich Reindeer (Zoo)
	(Cross-validated)	
**Blind Test (based on the protocol)**		
Racing / zoo (Step 1)	100.0%	6/6 Zoo
Free ranging / zoo males (Step 2)	96.0%	2/2 Zoo
Free ranging / zoo females (Step 2)	90.0%	3/4 Zoo
**Additional Classifications**		
All groups	65.0%	6/6 Zoo
All groups’ males	72.7%	2/2 Zoo
Racing / free ranging	88.1%*	6/6 Free ranging

Additionally, to further explore the reliability of our discriminant equations, we also applied some of our functions that were not included in the proposed protocol. In these cases, as demonstrated in the lower part of Table 5, all six specimens where classified as zoo (when the zoo group was present in the comparison). In the DFA equation that did not include the “zoo” group (i.e., from the DFA focusing on the pair comparison between racing and free ranging individuals), all six specimens were classified as “free ranging” (as opposed to racing ones).

## Discussion

This study presented the first application of the VERA protocols on the entheses of remains of a zoological collection, identifying distinct variation across reindeer of distinct activity groups (zoo, racing, and free ranging animals). Our high classification accuracy rates (Table 3) can be attributed to our strategy for selecting entheses based on each group’s habitual activities and the use of the experimentally validated approach VERA (Karakostis and Lorenzo 2016; Karakostis and Harvati 2021; Karakostis 2022), which includes the use of multivariate statistical approaches for reconstructing frequent muscle coordination. These results are in line with previous experimental applications of VERA on laboratory rats, mice, and turkeys (Karakostis et al. 2019a; 2019b; Karakostis and Wallace 2023; Castro et al. 2022).

When analysing all three groups and both sexes together (Fig. 2A; Table 3), there were clear differences between racing and zoo reindeer, but both of these groups extensively overlapped with free ranging animals. Nevertheless, when focusing only on the males (Fig. 2B; Table 3), most free ranging reindeer showed a distinctive pattern as well, exhibiting only partial overlapping with the racing group. This result highlights the importance of controlling for the effects of sexual dimorphism when reconstructing physical activity based on entheseal morphology (e.g., see Schrader 2019). Comparing groups in pairs provided even clearer results, also revealing partial — albeit more limited — overlapping between racing and free ranging animals (Fig. 2F). This similarity between certain racing and free ranging animals seems to be consistent with the similar physical activities between these two groups (see Fig. 1). In contrast, the activities of the zoo animals differ substantially from all other specimens (Fig. 1). As a result, when comparing all three groups within the comparative framework of PCAs or DFAs (e.g., Table 3; Fig. 2A and B), it is possible that the substantial differences between the zoo (sedentary) and the other two less sedentary groups are the main factors influencing the principal axes of variance or group differentiation. This may cause some of the distinctions between the free ranging and racing groups (more active groups) to be obscured.

Importantly, the DFAs of this study have led to identifying a specific sequence of discriminating equations, which can be used to classify reindeer into activity groups with a cross-validated classification rate that ranges from 88 to 100% (see step-by-step instructions in Online resources; pg. 1–4 and Tables 1–6). More specifically, a seemingly perfect (100%) classification rate was reached when comparing between sedentary reindeer (zoo) and reindeer exercising intense activities (racers), especially when taking sex into account (Table 3). This is further confirmed by the results of our blind test, which led to a correct activity reconstruction for five out of six zoo specimens from Munich (i.e., from a different population and geochronological context) (Table 1). It is worth noting that the single misclassified specimen was correctly assigned using other function equations (see Table 5), encouraging future research to consider applying several of the functions proposed in this thesis (See protocol, Online resources pg. 1–4). Similarly, very high classification rates were possible when distinguishing free ranging reindeer (i.e., those involved in natural activities for that species) from those with either very limited (92–100%) or highly intense physical behaviour (88%). These findings support the future use of our approach and step-by-step protocol (Online resources pg. 1–4) for assessing physical activity in reindeer from bioarchaeological contexts (but see subsection below).

The entheses of certain muscles or ligaments showed considerable differences across groups in both the PCAs and the DFAs of this study. The muscle *teres major*, in addition to being involved in shoulder and humeral movement, contributes to the differentiation between free ranging specimens and all other reindeer. This might reflect the habitual performance of activities in the wild, such as digging in the snow for lichen (Fig. 1). During the latter activity, this muscle serves to draw the limb towards the midline of the body (König et al. 2007) despite significant resistance from the snow (which can reach significant depths in the Artic). The entheses of the *supraspinatus, flexor profundus* (ulnar head), and distal radio-ulnar ligament also appear to drive the observed variation between free ranging reindeer and the zoo group, most likely due to their contribution to muscle interactions for digging. On the other hand, comparing racing and free ranging animals (using the reconstructed synergy of muscles *supraspinatus, subscapularis, deltoideus* and *infraspinatus)* was better able to capture the differences between these two groups. These muscles are involved in the propelling movement of the shoulder (extension–flexion) performed by reindeer while running (König et al. 2007; Wareing et al. 2011), an activity expected to have been more intense in the “athletes” of our group (Fig. 1).

### Effects of subspecies, biological sex, age, and body size

The presence of two different subspecies in our sample (i.e., *R.t.fennicus* and *R.t.tarandus*) has been discussed in previous studies on the same reindeer sample (Niinimäki and Salmi 2016; Pelletier et al. 2020; Salmi et al. 2020; Niinimäki et al. 2021). We assessed whether the inclusion of these subspecies may have affected our observed patterns using additional analyses (Online resources 7 & 8). These showed that the distributions of the *R.t. tarandus* and *R.t. fennicus* within the free ranging group are very similar (Online resource 7), while repeating our activity comparisons within the same subspecies did not considerably change our results and interpretations (Online resource 8).

Across our results, the effects of sexual dimorphism were evident and clearly influenced the observed differentiation between activity groups (Table 3; Fig. 2). Consequently, in most of our analyses, analysing females and males separately appeared to increase the observed variability by activity group.

As shown in the Results, our Spearman’s correlation tests (Table 4) found significant correlations between body weight and some of the patterns (PC scores) observed in this study. The highest correlation was found when analysing both sexes together. This is clearly due to the fact that body size is sexually dimorphic in reindeer, further supporting our strategy to include sex-specific analyses in this study (Puputti and Niskanen 2008). The only two sex-specific analyses where weight was significantly correlated with PC scores were those comparing racing to zoo males and free ranging to zoo females. As far as the former comparison is concerned, it appears that the castrated status of some racers, as opposed to the non-castrated zoo males, may have resulted in an increase in weight and bulk, as it is frequently reported for reindeer (e.g., Niinimäki and Salmi 2016). In addition, the preference for selecting larger sized reindeer for racing competitions results in a bias towards larger body size.

The factors that contribute to the significant body size correlations observed in free ranging and zoo females (Table 4) are not clear. In general, due to their adaptation to changing food availability, free ranging females experience greater body mass fluctuations (Fauchald et al. 2004; Chan-McLeod et al. 1999; Ryg and Jacobsen 1982). However, whether these body mass adaptations in free ranging reindeer are the underlying cause of the correlation significance is unknown. Finally, while free ranging females have a higher average body weight than their zoo counterparts, the difference is not statistically significant based on an independent t-test (p > 0.05).

Finally, in this study, we chose to focus on young to late adults with no signs of bone deterioration (arthritis, exostosis, etc.) or pathologies in this study. Pathologies in more senile individuals have been shown to affect entheses by potentially distorting their robustness and, as a result, the interpretability of activity signals (Foster et al. 2014; Karakostis and Lorenzo 2016; Karakostis et al. 2017). Juveniles were also excluded as they were underrepresented in the sample, which prevented us from establishing a juvenile subgroup to test the effects of age on entheseal development. Although juvenile bones are more plastic and responsive to biomechanical pressures than adult bones, previous work has reported that entheseal robusticity is not as prominent (Foster et al. 2014).

### Applicability to the archaeological record and limitations

Our findings suggest that our VERA-based approach constitutes a highly valuable tool for reconstructing physical activity and potential domestication practices in reindeer. The sample used in this study, albeit modern, is broadly comparable to samples deriving from documented archaeological practices. For instance, the corralling of reindeer (Aronsson 1991; Andersen 2011; Pelletier et al. 2020) can arguably be compared to the conditions of our sample’s zoo reindeer. Furthermore, the use of reindeer for labour (used as draft or riding animals) broadly corresponds to our sample’s racing reindeer (Niinimäki and Salmi 2016; Salmi et al. 2020). On this basis, we believe that the comparative approach proposed in this study can provide valuable information on the habitual activities of reindeer deriving from bioarchaeological context. Moreover, for further increasing the accuracy of activity reconstructions in reindeer, future studies may integrate our methodology with other proposed lines of evidence (Niinimäki and Salmi 2016; Pelletier et al. 2020; 2021; Salmi et al. 2020; Salmi and Niinimäki 2021; Niinimäki et al. 2021), such as other skeletal activity markers (e.g., cross-sectional geometry; Pelletier et al. 2021; Profico et al. 2021; Niinimäki and Salmi 2021; Niinimäki et al. 2021) animal domestication artefacts (e.g., Anderson et al. 2019), corralling evidence (e.g., Andersen 2011; Gaunitz et al. 2018; Outram et al. 2009), and pollen data (e.g., Aronsson 1991).

However, it must be highlighted that unlike modern samples with known life histories, the exact circumstances under which reindeer from an archaeological context were kept or used can often be far more complex. For instance, according to ethnographic accounts and archaeological evidence, female reindeer were corralled for the purposes of obtaining milk as well as their as decoy animals to attract males of free ranging herds (Helskog and Indrelid 2011; Anderson et al. 2019). On the other hand, male individuals were mostly used as draft animals (Andersen 2011; Anderson et al. 2019). Nevertheless, the exact level of mobility in such past reindeer is unknown and the prospect of them being able to graze in large pasture areas cannot be dismissed. Furthermore, this sample’s racing reindeer were engaged in a specialized activity that is very similar to that of draft reindeer. However, the load draft reindeer were subjected to (ca 250 kg) greatly differs from the weight of the racing reindeer had to pull (≥ 65 kg) (Niinimäki and Salmi 2016). Future studies could address this issue by analysing a sufficient sample size of draft individuals and compare them to the racing group.

Importantly, bone preservation is a well-known limitation in archaeology, which can undoubtedly affect attempts to reconstruct activity based on the functional morphology of skeletal remains. To accurately measure entheses and reconstruct their biological features (i.e., adult age, sex and weight) complete or nearly complete bones are required. Future studies could explore alternative muscle synergies that are engaged in the movements described in Table 2. These synergies could involve, for example, entheses in the distal humerus or the metapodia, in a reference sample which would help address the issue of preservation (fragmentation) by providing more options for application of our proposed approach. Finally, sex is an important factor that would ensure maximum classification accuracy, and studies have been able to successfully sex reindeer bones based on osteometric analyses that do not necessarily involve the entire bone (Weinstock 2000; van den Berg et al. 2022). Despite this, our analyses showed that correct classification can occur without information of sex with an 88% accuracy in the free ranging and zoo groups and 100% in the racing and zoo groups.

## Conclusions

This study presented a new and reliable approach for reconstructing habitual activity in reindeer based on their skeletal remains and relying on the experimentally validated VERA protocols (Karakostis and Lorenzo 2016; Karakostis and Harvati 2021; Karakostis 2022; and references therein). Following blind analytical conditions, we demonstrated that the proposed approach can accurately distinguish across three different activity groups (racing, free ranging, and zoo animals). These findings provide an original tool for identifying domestication-related activities in reindeer from bioarchaeological contexts. In the future, we believe that the integration of our approach with other proposed lines of evidence (e.g., pollen data, corralling evidence, or cross-sectional geometry of lone bones) and the expansion of our approach to other faunal species can contribute to establishing a novel framework for evaluating potential domestication hotspots in the archaeological record.

### Supplementary Information

Below is the link to the electronic supplementary material.

Supplementary file1 (DOCX 1346 KB)

## Data Availability

The data produced in this present study are available from the corresponding author upon reasonable request.

## Declarations

### Animal welfare statement

The primary sample originates from the Biodiversity Unit at the University of Oulu. The skeletal remains were obtained from reindeer that were not subjected to experimental processes or were interacted with during their lifetimes. No harm befell animals for the purposes of this study. The assemblage was accumulated through prior projects under the ERC Starting Grant 756431 and Academy of Finland projects 285774 and 308322. Research findings were published in various publication venues, including the Journal of Archaeological and Anthropological Sciences. The Biodiversity Unit at the University of Oulu is officially responsible for studying and curating this material following established ethical guidelines and has granted approval for its use in this study. The secondary sample originates from the State Collection of Anthropology and Palaeoanatomy (SNSB) in Munich, Germany. These remains also originate from reindeer that were not engaged in experimental processes or were interacted with during their lifetimes. No harm came to the animals for the purposes of this study. The SNSB is officially responsible for the material's study and curation following established ethical guidelines and has provided approval for its utilization.
