# Supplementary material for: Reconstructing patterns of domestication in reindeer using 3D muscle attachment areas
Source: Archaeol Anthropol Sci. 2023 Dec 29;16(1):19. doi: 10.1007/s12520-023-01910-5 (PMC10756864; doi:10.1007/s12520-023-01910-5)
Supplement: Supplementary file 1 — Supplementary file1 (DOCX 1346 KB) [file 12520_2023_1910_MOESM1_ESM.docx]

# Reconstructing patterns of domestication in reindeer using 3D muscle attachment areas: Online Resources

Christina Siali^1*^, Sirpa Niinimäki^2^, Katerina Harvati^1,3*^ & Fotios Alexandros Karakostis^1,3*^

1 - Palaeoanthropology, Senckenberg Centre for Human Evolution and Palaeoenvironment, Institute for Archaeological Sciences, Eberhard Karls University of Tübingen, Tübingen, Germany · 2 - Archaeology, History, Culture and Communication Studies, Faculty of Humanities, University of Oulu, Oulu, Finland · 3 - DFG Centre of Advanced Studies ‘Words, Bones, Genes, Tools’, Eberhard Karls University of Tübingen, Tübingen, Germany.

## *Correspondence: [christina.siali@uni-tuebingen.de](mailto:christina.siali@uni-tuebingen.de)

# 1.Methodological Suggestions

Considering the originally high classification percentages provided by our DFAs, we recommend the use of our approach for reconstructing habitual activity in reindeer from archaeological or other modern samples. The application of the method proposed here would require the following steps:

1. Measurement of the entheseal surface in a 3D environment using the VERA protocols (Karakostis and Lorenzo 2016; Karakostis and Harvati 2021; Karakostis 2022) and subsequent size adjustment of the measurements using geometric mean.

2. Information about weight, sex, and adult age status of the individuals should be available or reconstructed (Morrison and Whitridge 1997; Puputti and Niskanen 2008; Pasda 2009).

3. Application of a discriminant function equation to assess the activity group membership of the reindeer under examination. In this context, we propose a series of discriminant function equations using :

**y = b_1_x_1_ + b_2_x_2_ + … + b_n_x_n_ + c**

where b represents unstandardized coefficients and c represents constants (Hardy 1993). The information needed for the parameters of the equations as well as the muscle combinations are listed in the supplementary material (Online resource 11 & 12). Following the numerous pair combinations presented in this study we propose the following sequence of steps, corresponding to the application of specific discriminant function equations (i.e., steps 1 and 2):

**Step 1:** Racing versus Zoo. As racing and zoo arguably represent the opposite extremes of activity (high versus low) in our sample, we suggest testing this combination first. In this case, either by analysing females and males together or separately (Online resources 1&2), our DFA achieved 100% of correct classification, therefore the use of either combination would be sufficient.

| **DFA** | **Group Classification % (Original/cross validated** | **Group Centroids** | | **Unstandardized coefficients** | | | |  |  |
| --- | --- | --- | --- | --- | --- | --- | --- | --- | --- |
|  |  | **Racing** | **Zoo** | **SUBSC** | **SUPRA** | **DELT** | **LIGAM** | **Constant** |  |
|  |  |  |  |  |  |  |  |  |  |
|  |  |  |  |  |  |  |  |  |  |
| Zoo – Racing / Raw | 100%/ 100 % | 5.538 | -1.69 | 0.008 | -0.001 | 0.006 | -0.005 | -4.655 |  |

**Online resource 1.** Classification percentages, group centroids, unstandardized coefficients^1^ and constants of the discriminant function analysis comparing **females and males of the zoo group against the males of the racing group** (all male sample).

| **DFA** | **Group Classification % (Original/cross validated** | **Group Centroids** | | **Unstandardized coefficients** | | |  |  |
| --- | --- | --- | --- | --- | --- | --- | --- | --- |
|  |  | **Racing** | **Zoo** | **DELT** | **FPD** | **LIGAM** | **Constant** |  |
|  |  |  |  |  |  |  |  |  |
|  |  |  |  |  |  |  |  |  |
| Zoo - Racing Males / Raw | 100%/ 100% | 2.168 | -3.469 | 0.007 | 0.015 | -0.009 | -8.669 |  |

**Online resource 2.** Classification percentages, group centroids, unstandardized coefficients^1^ and constants of discriminant function analyses involving **only males of the zoo and racing groups**.

**Step 2:** Comparing the result with free ranging. This step is dependent on the results of the function equation applied in Step 1. If the resulting discriminant score indicates that the undocumented individual (s) is classified with the zoo group, then the possibility that the specimen was actually a free ranging (rather than zoo) individual should be additionally tested following Step 2a (below). In contrast, if the results of the first step (Step 1) indicate that the reindeer under examination might be a racing individual, then the function equation of Step 2b (below) must be followed (associating specimens with either racing or free-ranging activities).

**2a.** Free-ranging vs Zoo: When comparing free-ranging vs zoo animals, it is highly recommended to use function equations based on males and females separately using raw measurements, to achieve classification results that reached 100% in males (Online resource 3) and 91% in females (Online resource 4). If biological sex cannot be estimated, both functions should be applied. In steps 1 and 2a, the analysis of muscles involved in the movement of both upper and lower limb is recommended (see Online resource 3 below).

| **DFA** | **Group Classification % (Original/cross validated** | **Group Centroids** | | **Unstandardized coefficients** | | | |  |  |
| --- | --- | --- | --- | --- | --- | --- | --- | --- | --- |
|  |  | **Free- ranging** | **Zoo** | **INFRA** | **DELT** | **TERES** | **FPUH** | **Constant** |  |
|  |  |  |  |  |  |  |  |  |  |
|  |  |  |  |  |  |  |  |  |  |
| Free ranging - Zoo Males / Raw | 100%/100% | 1.123 | -3.146 | 0.002 | 0.003 | 0.007 | 0.001 | -8.259 |  |

**Online resource 3.** Classification percentages, group centroids, unstandardized coefficients^2^ and constants of Discriminant function analyses involving **only males of the zoo and free- ranging groups**.

| **DFA** | **Group Classification % (Original/cross validated** | **Group Centroids** | | **Unstandardized coefficients** | | |  |  |
| --- | --- | --- | --- | --- | --- | --- | --- | --- |
|  |  | **Free- ranging** | **Zoo** | **TERES** | | **LIGAM** | **Constant** |  |
|  |  |  |  |  |  |  |  |  |
|  |  |  |  |  |  |  |  |  |
| Free - Zoo Females / Raw | 91.3%/91.3% | 0.722 | -1.65 | 0.01 | 0.007 | | -4.852 |  |

**Online resource 4.** Classification percentages, group centroids, unstandardized coefficients^1^ and constants of Discriminant function analyses involving **only females of the zoo and free- ranging groups.**

**2b:** Racing vs Free-ranging reindeer. For this comparison, the DFA function equation based on size adjusted measurements and pooled sexes (both females and males together) seemed to work best, providing an 88.1% correct classification rate (Online resource 5). An 80% accuracy was provided when only males were analysed using the muscle entheses of the upper front limb (Online resource 6). Nevertheless, it is worth noting that, when only males were analysed, 100% of racing individuals were correctly classified (8/8 racing) (Online resource 6)

| **DFA** | **Group Classification % (Original/cross validated)** | **Group Centroids** | | **Unstandardized coefficients** | |  |  |
| --- | --- | --- | --- | --- | --- | --- | --- |
|  |  | **Free- ranging** | **Racing** | **INFRA** | **DELT** | **Constant** |  |
|  |  |  |  |  |  |  |  |
|  |  |  |  |  |  |  |  |
| Free- Racing / GM | 90.5%/88.1% | -0.396 | 1.682 | 3.995 | 2.204 | -7.849 |  |

**Online resource 5.** Classification percentages, group centroids, unstandardized coefficients^1^ and constants of Discriminant function analyses **involving the females and males of the free ranging group and the males of racing group** (all male sample) , using size adjusted (GM) measurements.

| **DFA** | **Group Classification % (Original/cross validated)** | **Group Centroids** | | **Unstandardized coefficients** | | | |  |  |
| --- | --- | --- | --- | --- | --- | --- | --- | --- | --- |
|  |  | **Free- ranging** | **Racing** | **INFRA** | **SUBSC** | **SUPRA** | **DELT** | **Constant** |  |
|  |  |  |  |  |  |  |  |  |  |
|  |  |  |  |  |  |  |  |  |  |
| Free - Racing Males / GM | 88%/80% | -0.571 | 1.212 | 4.828 | 0.314 | 0.503 | 2.652 | -10.899 |  |

**Online resource 6.** Classification percentages, group centroids, unstandardized coefficients^[[1]](#footnote-1)^ and constants of Discriminant function analyses involving **only males of the free ranging and racing groups**, using size adjusted (GM) measurements.

# 2. Subspecies

The presence of two reindeer subspecies in the sample, as well as their potential effect on group differentiation patterns, was explored further using PCA. The two subspecies, *R.t.tarandus* and *R.t.fennicus,* are present both in the zoo group and free ranging groups (Niinimäki and Salmi 2016; Pelletier et al. 2020; Salmi et al. 2020; Niinimäki et al. 2021; Salmi and Niinimäki 2021; Pelletier et al. 2021, 2022). However, *R.t.tarandus* constitutes 16% of the overall sample in the free-ranging group (84% being *R.t.fennicus*), while it constitutes 50% of the total sample in the zoo group (with remaining 50 % *R.t.fennicus* and a hybrid). To address this disparity, we first explored subspecies variation within the same activity group (Online resource 7), finding extensive overlapping between the two subspecies.

Subsequently, we performed a pair comparison of the free ranging and zoo groups within the same subspecies to assess if the study’s observations of activity differences change when eliminating subspecies variation (Online resource 8). To maintain an adequate sample size with minimum 5 individuals per group, we analysed *the R.t. fennicus* and *R.t tarandus* free ranging females in Online resource 7. Since it was clear from both the PCA’s and DFA reported in the paper (Figure 2 & Table 3) that sexual dimorphism played a role in the differentiation of the activity groups, males were excluded from PCA. Unfortunately, the sample size of male free ranging *R.t.tarandus* and *R.t.fennicus* was not adequate for a separate analysis involving only males. Similarly, in Supplementary Figure 2 , due to sample size restrictions we analysed only the *R.t.tarandus* females of the free ranging and zoo groups.

In both Online resources 7 and 8, it appears that: 1) variation within the free ranging group does not display any distinctive trends between the *R.t. tarandus* and *R.t. fennicus* females, and 2) Subspecies variation had no effect on activity group differentiation or entheseal patterns since activity patterns remained similar even within the same subspecies.


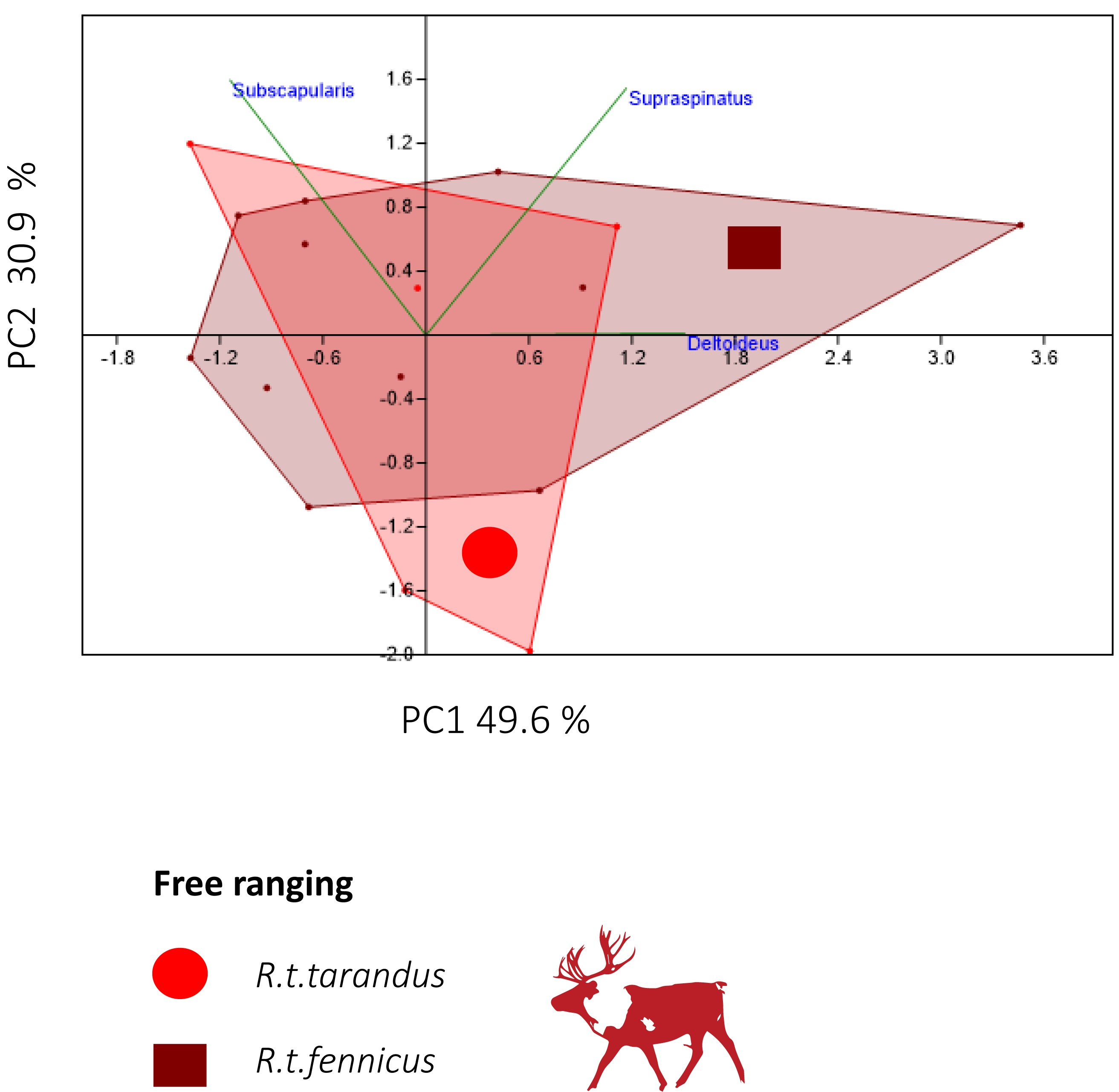


|  |  |  | Factor Loadings | | |
| --- | --- | --- | --- | --- | --- |
| **PC** | **Eigen Value** | **% of Variance** | **SUB** | **SUPRA** | **DELT** |
| 1 | 1.49094 | 49.698 | -0.513 | 0.5251 | 0.6788 |
| 2 | 0.92864 | 30.955 | 0.7183 | 0.6957 | 0.0050 |

**Online Resource 7**. PCA on the free ranging group with R.t.tarandus females presented in bright red (circle) and R.t.fennicus females in dark red (square). Eigen values, factor loadings and percentage of variance are listed below the figure.


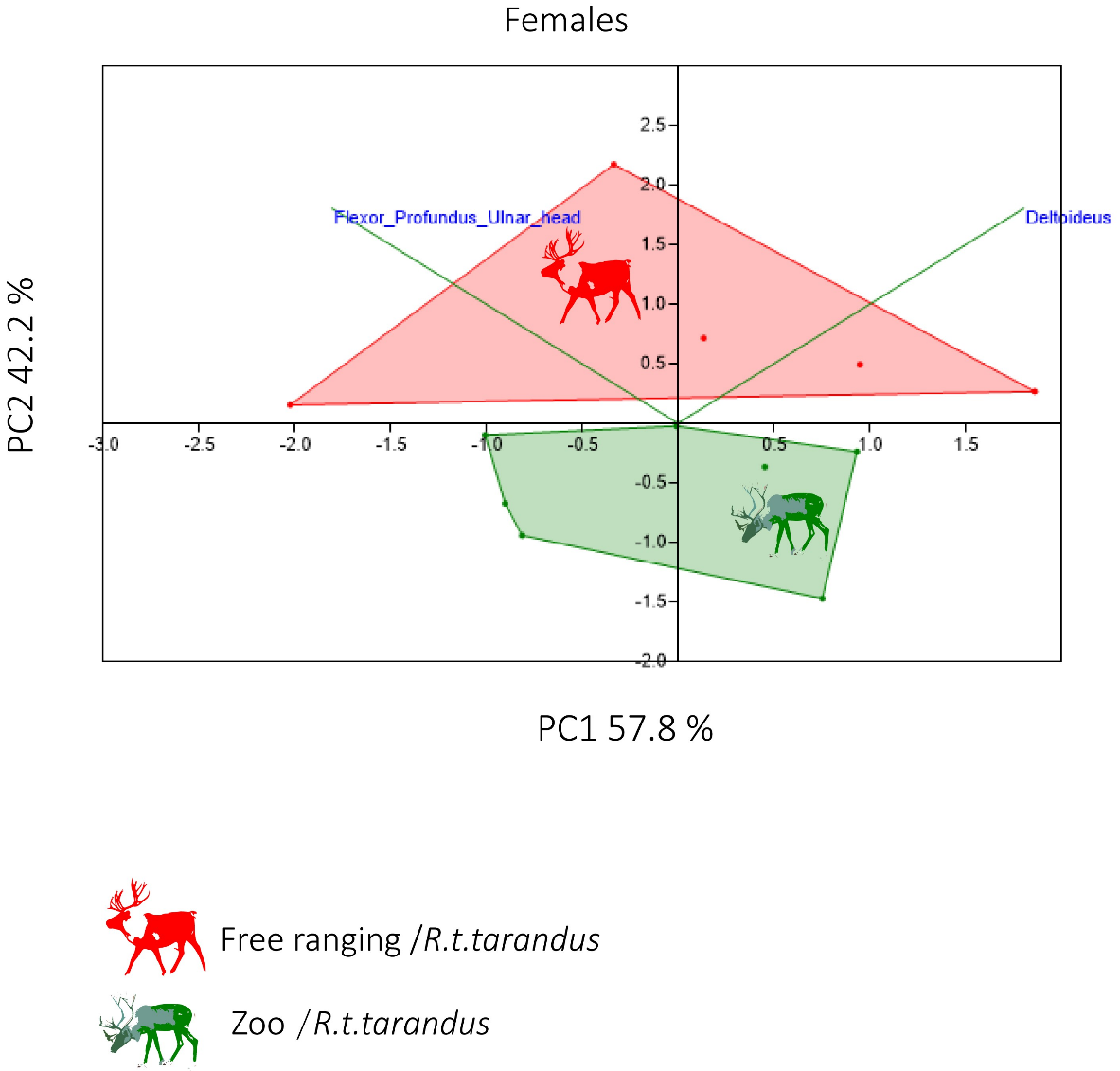


|  |  |  | Factor Loadings | |
| --- | --- | --- | --- | --- |
| **PC** | **Eigen Value** | **% of Variance** | **FPUH** | **DELT** |
| 1 | 1.5628 | 57.814 | 0.7071 | 0.7071 |
| 2 | 0.843718 | 42.186 | -0.7071 | 0.7071 |

**Online Resource 8**. PCA of the pair comparison of the free ranging (in red) and Zoo (in green) females involving only *R.t.tarandus* individuals, eigenvalues, factor loadings and % of cariance, are listed below the figure.

# 3. Additional PCAs and PCA statistics

## PCA statistics of Figure 2 in Results

|  |  |  |  |  |  | **Factor Loadings** |  |  |  |  |  |  |
| --- | --- | --- | --- | --- | --- | --- | --- | --- | --- | --- | --- | --- |
|  | **PC** | **Eigen Value** | **% of Variance** |  | **INFRA** | **SUB** | **SUPRA** | **DELT** | **T MAJOR** | **RAU LIG** | **FDP** | **FPUH** |
|  | PC1 | 4.68725 | 58.591 |  | 0.2555 | 0.3613 | 0.2816 | 0.3863 | 0.3997 | 0.3929 | 0.3407 | 0.3814 |
| A | PC2 | 1.05813 | 13.227 |  | 0.6869 | -0.07391 | 0.5943 | -0.11 | -0.2473 | -0.05764 | -0.2479 | -0.1775 |
|  | PC3 | 0.683106 | 8.5388 |  | 0.2154 | 0.3299 | -0.248 | -0.4874 | -0.2695 | 0.3196 | 0.5299 | -0.3002 |
|  | PC1 | 2.36857 | 47.371 |  |  |  | 0.4097 |  | 0.5049 | 0.4874 | 0.4227 | 0.4013 |
| B | PC2 | 1.00583 | 20.117 |  |  |  | 0.3948 |  | 0.01766 | -0.417 | -0.5217 | 0.6307 |
|  | PC3 | 0.746284 | 14.926 |  |  |  | 0.7278 |  | -0.4557 | 0.3102 | -0.1642 | -0.3735 |
| C | PC1 | 2.13484 | 53.371 |  |  |  |  | 0.5315 | 0.5784 | 0.4634 |  | 0.4102 |
|  | PC2 | 0.95496 | 23.874 |  |  |  |  | -0.5117 | -0.35 | 0.4635 |  | 0.633 |
|  | PC3 | 0.599337 | 14.983 |  |  |  |  | 0.1347 | 0.00597 | -0.7426 |  | 0.656 |
|  | PC1 | 2.15504 | 71.835 |  |  |  |  | 0.5705 | 0.6362 | 0.5194 |  |  |
| D | PC2 | 0.637308 | 21.244 |  |  |  |  | -0.6074 | -0.0989 | 0.7882 |  |  |
|  | PC3 | 0.207649 | 6.9216 |  |  |  |  | 0.5528 | -0.7652 | 0.33 |  |  |
| E | PC1 | 1.74366 | 87.183 |  |  |  |  | 0.7071 | 0.7071 |  |  |  |
|  | PC2 | 0.256344 | 12.817 |  |  |  |  | 0.7071 | -0.7071 |  |  |  |
|  | PC1 | 1.84304 | 46.076 |  | 0.5255 | 0.3153 | 0.6431 |  |  | 0.4591 |  |  |
| F | PC2 | 1.18009 | 29.502 |  | -0.5772 | 0.7449 | -0.1173 |  |  | 0.3133 |  |  |
|  | PC3 | 0.726704 | 18.168 |  | 0.01557 | -0.3979 | -0.4052 |  |  | 0.823 |  |  |
|  |  |  |  |  |  |  |  |  |  |  |  |  |

**Online resource 9.** Eigen values, factor Loadings and variance percentages of the first three principal components of PCA of Figure 2 in Results. The asterisk * denotes the use of size adjusted (GM) measurements.

|  |  | **Group Centroids** | | | **Unstandardized coefficients** | | | | | | |
| --- | --- | --- | --- | --- | --- | --- | --- | --- | --- | --- | --- |
| **Discriminant function analyses** | **Group Classification % (Original/cross validated** | **Free- ranging** | **Racing** | **Zoo** | **INFRA** | **SUBSC** | **SUPRA** | **DELT** | **WEIGHT** | **Constant** | **Box's M Sign** |
| **Humeral Entheseal Surfaces (INFRA, SUPRA, SUBSC, DELT)** | | | | | | | | | | | |
| All groups/ Raw | 73%/65.1% | 0.313 | 1.789 | -1.192 | -0.002 | 0.001 | -0.003 | 0.004 | -0.002 | -2.07 | 0.000* |
| All groups/ GM | 66.7%/58.7% | 0.078 | 2.136 | -0.94 | 2.953 | 0.913 | 2.185 | 2.637 |  | -10.638 | 0.00* |
| Racing - Zoo/ Raw | 100%/100% |  | 3.709 | -1.413 | 0 | 0.005 | -0.005 | 0.007 |  | -4.623 | 0.004* |
| Racing - Zoo/ GM | 96.6%/96.6% |  | 2.232 | -0.85 |  |  |  | 2.146 |  | -4.998 | 0.232 |
| Free- Racing / Raw | 85.7%/83.3% | -0.36 | 1.531 |  | 0.003 | -0.006 | -0.009 | 0.004 |  | 0.172 | 0.02* |
| **Free- Racing / GM** | **90.5%/88.1%** | **-0.396** | **1.682** |  | **3.995** |  |  | **2.204** |  | **-7.849** | **0.414** |
| Free - Zoo / Raw | 78.2%/70.9% | 0.641 |  | -1.037 | -0.008 | 0.005 | 0.003 | 0.003 | -0.002 | -2.476 | 0.00* |
| Free - Zoo / GM | 70.9%/67.3% | 0.465 |  | -0.752 |  |  | 3.122 | 2.177 |  | -7.46 | 0.129 |
| All groups- Male/ Raw | 75.8%/72.7% | 0.714 | 0.849 | -2.365 | 0.002 | 0.005 | -0.002 | 0.005 |  | -6.088 | 0.084 |
| All groups- Male/ GM | 84.4%/69.7% | 0.016 | 1.682 | -1.716 | 3.171 | 1.016 | 4.054 | 3.246 |  | -14.642 | 0.009* |
| Free - Zoo Males / Raw | 96%/96% | 0.959 |  | -2.038 | -0.002 | 0.006 | 0 | 0.004 |  | -5.546 | 0.157 |
| Free - Zoo Males / GM | 96%/86% | 0.895 |  | -1.901 | 4.458 | 3.729 | 9.136 | 5.13 |  | -26.254 | 0.688 |
| Free - Zoo Females / Raw | 90%/90% | 0.606 |  | -0.792 |  |  |  | 0.031 |  | -9.279 | 0.056 |
| Free - Zoo Females /GM | 60%/46% | -0.401 |  | 0.524 | 4.491 | 0.428 | -1.356 | -0.498 | 0.003 | -1.369 | 0.00* |
| Free - Racing Males / Raw | 76%/60% | 0.505 | -1.072 |  | -0.001 | 0.009 | 0.008 | -0.003 |  | -2.459 | 0.141 |
| **Free - Racing Males / GM** | **88%/80%** | **-0.571** | **1.212** |  | **4.828** | **0.314** | **0.503** | **2.652** |  | **-10.899** | **0.02*** |
| Zoo - Racing Males / Raw | 100%/100% |  | 1.895 | 1.895 |  |  |  | 0.006 |  | -5.026 | 0.292 |
| Zoo - Racing Males / GM | 93.8%/93.8% |  | 1.342 | -1.342 |  |  |  | 2.002 |  | -5.403 | 0.275 |

# 4. Additional DFAs and DFA statistics

**Online resource 10.** Group Classification percentages, group centroids, unstandardized coefficients, and constants of DFAs of all groups, sexes and data combinations using four entheseal surfaces of the humerus (*subscapularis, infraspinatus*, *supraspinatus, deltoideus*) . Box’s M with significant p-values (denoted with asterisk *) were run again using separate groups covariance matrix . In bold are the DFA listed in Table 3 of the Results.

|  |  | **Group Centroids** | | | **Unstandardized coefficients** | | | | | | | |  |  |  |
| --- | --- | --- | --- | --- | --- | --- | --- | --- | --- | --- | --- | --- | --- | --- | --- |
| **Discriminant function analyses** | **Group Classification % (Original/cross validated** | **Free- ranging** | **Racing** | **Zoo** | **INFRA** | **SUBSC** | **SUPRA** | **DELT** | **TERES** | **FPD** | **FPUH** | **LIGAM** | **WEIGHT** | **Constant** | **Box's M Sign** |
| **All Humeral and Radioulnar entheseal surfaces** | | | | | | | | | | | | | | | |
| All groups/ Raw | 68%/ 66% | 0.244 | 1.146 | -1.37 |  |  |  | 0.002 | 0.004 |  |  | -0.001 |  | -2.818 | 0.109 |
| **All groups/ GM** | **74%/ 70%** | **0.227** | **1.306** | **-1.437** |  |  | **-0.893** | **0.983** | **3.156** |  |  | **-2.908** |  | **-0.767** | **0.893** |
| Free - Zoo / Raw | 88.1%/85.7% | 0.609 |  | -1.523 |  | 0.007 |  |  | 0.009 |  |  | -0.003 | -0.024 | -1.374 | 0.408 |
| Free - Zoo / GM | 88.1%/85.7% | -0.545 |  | 1.363 |  |  |  |  | -3.194 | 0.499 | 1.737 | 2.43 |  | -3.467 | 0.956 |
| Free- Racing / Raw | 84.2%/81.6% | -0.381 | 1.427 |  |  |  | -0.006 |  |  | 0.004 | 0.001 | -0.007 |  | -0.953 | 0.061 |
| Free- Racing / GM | 84.2%/81.6% | 0.352 | -1.319 |  |  |  | 3.817 |  | 1.946 |  | 0.863 | 5.041 | -0.13 | -8.97 | 0.9 |
| **Racing - Zoo/ Raw** | **100%/100 %** |  | **5.538** | **-1.692** |  | **0.008** | **-0.001** | **0.006** |  |  |  | **-0.005** |  | **-4.655** | **0.311** |
| Racing - Zoo/ GM | 100%/95% |  | 2.011 | -1.341 |  |  | -1.627 | 1.288 | 2.659 |  |  | -4.82 |  | 1.806 | 0.729 |
| All groups- Male/ Raw | 85.2%/77.8% | 1.068 | 0.074 | -3.11 |  | 0.003 | 0.003 | 0.003 | 0.007 |  |  | 0 |  | -7.922 | 0.061 |
| All groups- Male/ GM | 77.8%/63% | 0.368 | 0.659 | -2.135 |  | -2.527 | 1.615 | 2.327 | 4.303 |  |  |  |  | -7.618 | 0.489 |
| **Free - Zoo Males / Raw** | **100%/100%** | **1.123** |  | **-3.146** | **0.002** |  |  | **0.003** | **0.007** |  | **0.001** |  |  | **-8.259** | **0.404** |
| Free - Zoo Males / GM | 94.7%/94.7% | 0.507 |  | -1.421 |  |  | 2.329 |  | 5.775 | -0.127 |  |  |  | -6.412 | 0.106 |
| **Free - Zoo Females / Raw** | **91.3%/91.3%** | **0.722** |  | **-1.65** |  |  |  |  | **0.01** |  |  | **0.007** |  | **-4.852** | **0.158** |
| Free - Zoo Females /GM | 78.3%/78.3% | 0.502 |  | -1.148 |  |  |  |  | 4.612 |  |  |  |  | -3.344 | 0.55 |
| Free - Racing Males / Raw | 77.3%/77.3% | 0.571 | -0.999 |  |  |  | 0.006 | -0.001 |  |  |  | 0.007 |  | -5.231 | 0.322 |
| Free - Racing Males / GM | 77.3%/72.7% | 0.621 | -1.087 |  |  | 4.646 | 6.148 | -0.031 | 3.879 |  |  | 4.403 |  | -14.142 | 0.357 |
| Zoo - Racing Males / Raw | 100%/100% |  | 2.168 | -3.469 |  |  |  | 0.007 |  | 0.015 |  | -0.009 |  | -8.669 |  |
| **Zoo - Racing Males / GM** | **100%/100 %** |  | **-1.723** | **2.757** | **6.788** | **9.61** |  |  |  |  |  | **7.11** |  | **-16.041** | **0.516** |

**Online resource 11.** Group Classification percentages, group centroids, unstandardized coefficients, and constants of DFAs of all groups, sexes and data combinations using four entheseal surfaces of the humerus and radio-ulna. In bold are the DFA listed in Table 3 of the Results.

# 5. References

Hardy MA, (1993) Regression with dummy variables. Sage Publications, Newbury Park

Karakostis A, Harvati K (2021) New horizons in reconstructing past human behavior: Introducing the “Tübingen University Validated Entheses-based Reconstruction of Activity” method. Evolutionary Anthropology: Issues, News, and Reviews n/a: https://doi.org/10.1002/evan.21892

Karakostis A, Lorenzo C (2016) Morphometric patterns among the 3D surface areas of human hand entheses. American Journal of Physical Anthropology 160:694–707. https://doi.org/10.1002/ajpa.22999

Karakostis FA (2022) Statistical protocol for analyzing 3D muscle attachment sites based on the “Validated Entheses-based Reconstruction of Activity” (VERA) approach. International Journal of Osteoarchaeology n/a: https://doi.org/10.1002/oa.3196

Morrison D, Whitridge P (1997) Estimating the Age and Sex of Caribou from Mandibular Measurements. Journal of Archaeological Science 24:1093–1106. https://doi.org/10.1006/jasc.1996.0189

Niinimäki S, Härkönen L, Puolakka H-L, et al (2021) Cross-sectional properties of reindeer long bones and metapodials allow identification of activity patterns. Archaeol Anthropol Sci 13:146. https://doi.org/10.1007/s12520-021-01337-w

Niinimäki S, Salmi A-K (2016) Entheseal Changes in Free-Ranging Versus Zoo Reindeer-Observing Activity Status of Reindeer: Entheseal Changes in Free-Ranging Versus Zoo Reindeer. Int J Osteoarchaeol 26:314–323. https://doi.org/10.1002/oa.2423

Pasda K (2009) Osteometry, and Osteological Age and Sex Determination of the Sisimiut Reindeer Population: (Rangifer tarandus groenlandicus). BAR Publishing

Pelletier M, Kotiaho A, Niinimäki S, Salmi A-K (2020) Identifying early stages of reindeer domestication in the archaeological record: a 3D morphological investigation on forelimb bones of modern populations from Fennoscandia. Archaeol Anthropol Sci 12:169. https://doi.org/10.1007/s12520-020-01123-0

Pelletier M, Kotiaho A, Niinimäki S, Salmi A-K (2022) Impact of selection and domestication on hindlimb bones of modern reindeer populations: Archaeological implications for early reindeer management by Sámi in Fennoscandia. Historical Biology 34:802–820. https://doi.org/10.1080/08912963.2021.1947268

Pelletier M, Niinimäki S, Salmi A-K (2021) Influence of captivity and selection on limb long bone cross-sectional morphology of reindeer. Journal of Morphology 282:1533–1556. https://doi.org/10.1002/jmor.21403

Puputti A-K, Niskanen M (2008) The estimation of body weight of the reindeer ( *Rangifer tarandus* L.) from skeletal measurements: preliminary analyses and application to archaeological material from 17th- and 18th-century northern Finland. Environmental Archaeology 13:153–164. https://doi.org/10.1179/174963108X343272

Salmi A-K, Niinimäki S (2021) Reindeer Physical Activity Patterns and Reconstruction of Feeding Behaviour: Implications for Reindeer Domestication and Human-Reindeer Interaction. In: Salmi A-K, Niinimäki S (eds) Archaeologies of Animal Movement. Animals on the Move. Springer International Publishing, Cham, pp 45–57

Salmi A-K, Niinimäki S, Pudas T (2020) Identification of working reindeer using palaeopathology and entheseal changes. Int J Paleopathol 30:57–67. https://doi.org/10.1016/j.ijpp.2020.02.001

1. INFRA= *Infraspinatus*, SUB= *Subscapularis*, SUPRA= *Supraspinatus*,DELT= *Deltoideus*, TERES= *Teres Major*, FPD= *Flexor profundus digiti*. FPUH= *Flexor profundus* ulnar head, LIG= Radio-ulnar ligament [↑](#footnote-ref-1)
